# Mechanistic Insights into the Neutralization of Cytotoxic Abrin by the Monoclonal Antibody D6F10

**DOI:** 10.1371/journal.pone.0070273

**Published:** 2013-07-29

**Authors:** Shradha Bagaria, Devasena Ponnalagu, Shveta Bisht, Anjali A. Karande

**Affiliations:** 1 Department of Biochemistry, Indian Institute of Science, Bangalore, Karnataka, India; 2 Molecular Biophysics Unit, Indian Institute of Science, Bangalore, Karnataka, India; Institut Curie, France

## Abstract

Abrin, an A/B toxin obtained from the *Abrus precatorius* plant is extremely toxic and a potential bio-warfare agent. Till date there is no antidote or vaccine available against this toxin. The only known neutralizing monoclonal antibody against abrin, namely D6F10, has been shown to rescue the toxicity of abrin in cells as well as in mice. The present study focuses on mapping the epitopic region to understand the mechanism of neutralization of abrin by the antibody D6F10. Truncation and mutational analysis of abrin A chain revealed that the amino acids 74–123 of abrin A chain contain the core epitope and the residues Thr112, Gly114 and Arg118 are crucial for binding of the antibody. *In silico* analysis of the position of the mapped epitope indicated that it is present close to the active site cleft of abrin A chain. Thus, binding of the antibody near the active site blocks the enzymatic activity of abrin A chain, thereby rescuing inhibition of protein synthesis by the toxin *in vitro*. At 1∶10 molar concentration of abrin:antibody, the antibody D6F10 rescued cells from abrin-mediated inhibition of protein synthesis but did not prevent cell attachment of abrin. Further, internalization of the antibody bound to abrin was observed in cells by confocal microscopy. This is a novel finding which suggests that the antibody might function intracellularly and possibly explains the rescue of abrin’s toxicity by the antibody in whole cells and animals. To our knowledge, this study is the first report on a neutralizing epitope for abrin and provides mechanistic insights into the poorly understood mode of action of anti-A chain antibodies against several toxins including ricin.

## Introduction

Abrin, a type II ribosome inactivating protein of plant origin comprises of catalytically active A chain harboring the RNA N-glycosidase activity and a lectin-like B chain responsible for binding and trafficking of the toxin in cells [1]. Abrin is known to be 75 times more potent than ricin with an LD_50_ of 2.8 µg/kg body weight in mice [2]. Despite the understanding of the molecular details of inhibition of protein synthesis by ricin and abrin [3], [4], [5], [6], the development of effective antidotes against these lethal potential bio-terror agents has been elusive. Substrate analogues of ricin have been tested [7], [8], [9] and found to be unsuitable owing to their toxicity and ineffective delivery inside cells [10]. However, monoclonal antibodies (mAbs) have been employed for the neutralization of several toxins [11], [12], [13]. Although many neutralizing antibodies have been reported against ricin [14], [15], [16], [17], [18], [19], the mAb D6F10 raised against abrin A chain (ABA) is the only known neutralizing antibody reported against abrin so far [20]. The antibody was shown to rescue cells and importantly also mice challenged with a lethal dose of abrin. However, the neutralizing epitope of the mAb D6F10 has not been identified. In contrast, few neutralizing epitopes corresponding to anti-ricin antibodies have been mapped [13], [21], [22], [23], [24]. Delineating epitopes of neutralizing antibodies is important to understand mechanisms involved in the immunoneutralization of toxins. Such studies would also provide better rationale for the design of vaccines against these lethal toxins [15].

Olsnes *et al*. reported that antibodies to any chain (either A or B) of ricin or abrin could neutralize the whole toxin [25]. This was later confirmed for many toxins [26], [27], [28] though the molecular mechanisms involved in neutralization by the anti-A chain antibodies still remain unclear. Some anti-ricin B chain antibodies have been proposed to sterically hinder the cell surface binding of the toxin [29], [30], [31]. However, antibodies against the toxin’s enzymatic A chain have had minimal effect on ricin binding and were proposed to interfere with intracellular toxin transport [31]. Studies on a potent antibody against ricin A chain (RTA) namely, R70 showed that the antibody interferes with attachment of ricin to receptors on the cell surface [13], [30]. Potential vaccine candidates for ricin and abrin have also been reported using the toxic A chain [32], [33]. These reports which suggest that both anti-A and anti-B chain antibodies might act by prevention of cell attachment of the toxin are unable to explain the reason for the higher efficiency of the anti-A chain antibodies in neutralization of ricin in comparison to anti-B chain antibodies.

The aim of this study was to delineate the neutralizing epitope on abrin and elucidate the mechanism of immunoneutralization of the toxin by the mAb D6F10. We demonstrate that the mAb D6F10 blocks the enzymatic activity of abrin A chain in a cell-free system by binding close to its active site. Furthermore, the internalization of the antibody along with abrin in cells possibly explains the rescue of cells from abrin’s cytotoxicity. The antibody might function intracellularly by altering the trafficking of the toxin inside cells or directly blocking its catalytic activity. Considering the earlier reports on anti-ricin antibodies [15], our findings may explain the mode of action and reason for higher efficiency of anti-ricin A chain antibodies.

## Results

### Mapping the Epitope of the mAb D6F10

**Figure 1:**
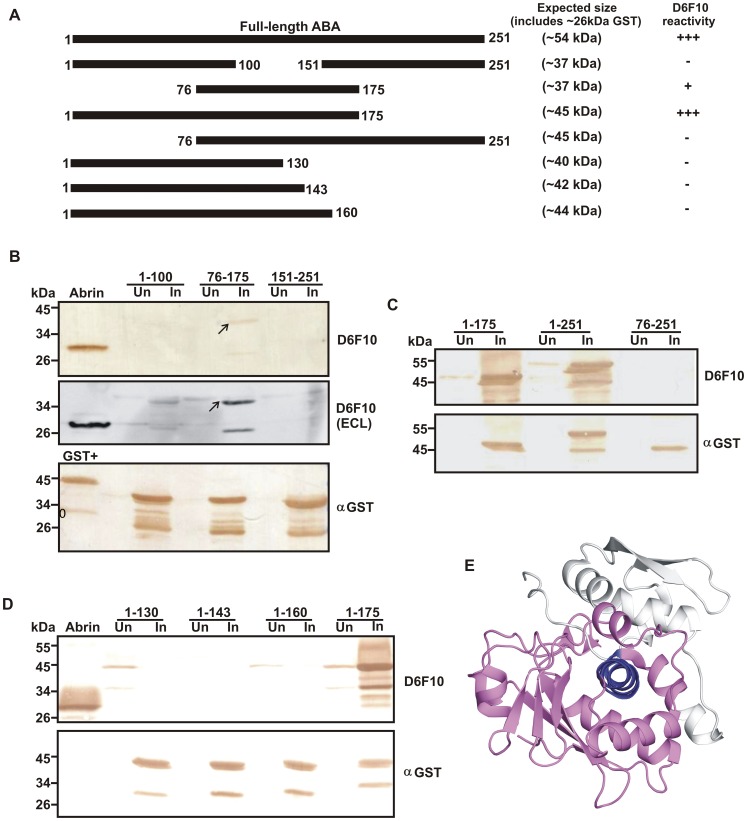
Amino acid sequence 1–175 of ABA is required for binding of the mAb D6F10. The uninduced (Un) and induced (In) samples of the recombinant ABA proteins expressed in *E. coli* were subjected to immunoblot analysis with mAb D6F10 or anti-GST antibody. (A) Schematic representation of the various truncated proteins of ABA with their expected molecular sizes, which includes the N-terminal GST tag (∼26 kDa). (B) Immunoblot analysis of the truncated ABA proteins spanning the amino acids 1–100, 76–175 and 151–251 with the mAb D6F10 using diaminobenzidine (DAB) or enhanced chemiluminescence (ECL). Weak binding of ABA 76–175 protein was observed with mAb D6F10. Native abrin (A chain of which is ∼30 kDa) and an in-house generated GST-perforin fusion protein (∼45 kDa) were used as positive controls for the mAb D6F10 and the GST blots respectively. (C) The recombinant protein ABA 1–175 bound the mAb D6F10 equivalent to the full-length ABA (1–251) but no binding was observed for ABA 76–251. (D) None of the C-terminal truncated forms of the protein ABA 1–175 namely, 1–130, 1–143 and 1–160 bound the mAb D6F10. (E) The structure of ABA representing the helix composed of amino acids 148–167 (in blue) forming the core of its structure. The amino acids 1–175 of ABA are colored pink and the truncated region of the ABA 1–175 protein spanning the amino acids 176–251 is colored grey. Truncation of the helix (in blue) might destabilize the structure and result in abrogation of antibody binding.

In order to map the epitope corresponding to the neutralizing mAb D6F10, overlapping truncated constructs spanning the entire length of the 251 amino acids long ABA were generated (Figure 1A). The expression of the desired GST-fusion proteins was confirmed using an anti-GST antibody (Figure 1B, 1C and 1D). Degraded products of the fusion proteins were also observed below the expected size bands (Figure 1A). When probed with the mAb D6F10, a faintly stained band observed with the truncated protein ABA 76–175 suggested that the amino acids within the stretch 76–175 of ABA form a part of the epitope (Figure 1B). Further, reduced binding of mAb D6F10 to the ABA 76–175 protein in comparison to the full-length native ABA suggested that the epitope corresponding to the mAb D6F10 might be discontinuous/conformation-dependent. An immunoblot developed with the more sensitive technique, enhanced chemiluminescence (ECL) also reconfirmed the earlier finding (Figure 1B). Immunoblot analysis of longer truncated forms of ABA showed that the mAb D6F10 bound the ABA 1–175 protein equivalent to full-length ABA, clearly suggesting that the epitope is discontinuous and lies within the first 175 amino acids of ABA (Figure 1C). Further truncations in the C-terminal region of ABA 1–175 resulted in loss of antibody binding (Figure 1D). *In silico* structural analysis of ABA revealed that the helix spanning amino acids 148–167 is present at the core of the ABA structure (Figure 1E). Therefore, truncation of the helix might destabilize the protein structure resulting in abrogation of antibody binding.

### Identification of ABA Residues that Constitute the Epitope of mAb D6F10

**Figure 2:**
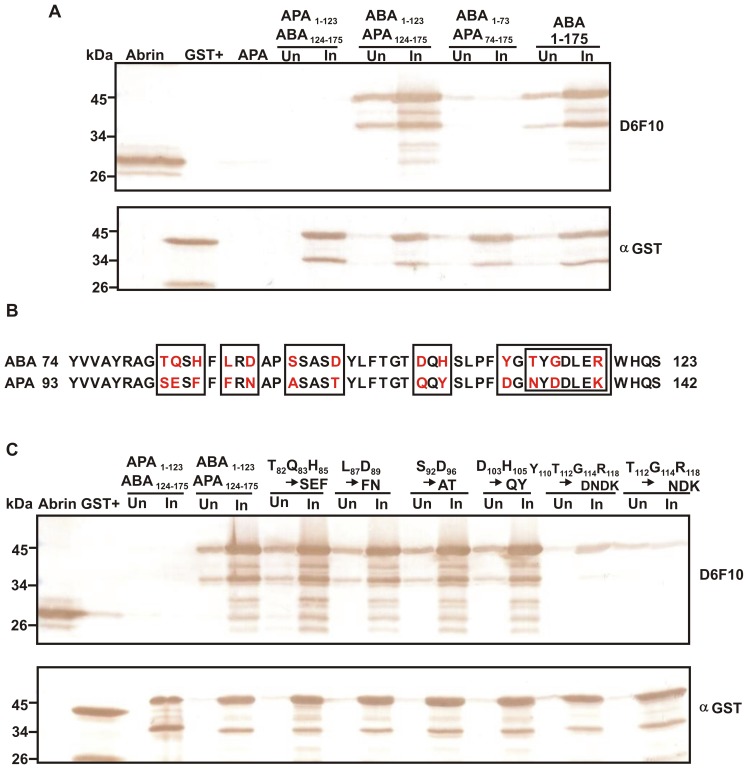
The core epitope corresponding to the mAb D6F10 includes the residues Thr112, Gly114 and Arg118 of ABA. The uninduced (Un) and induced (In) samples of the chimeric proteins (∼45 kDa) of ABA and APA A chain were subjected to immunoblot analysis with mAb D6F10 or anti-GST antibody. (A) The recombinant protein ABA_1–123_APA_124–175_ bound the mAb D6F10 whereas no binding was observed with the proteins APA_1–123_ABA_124–175_ and ABA_1–73_APA_74–175_. (B) Sequence alignment of the amino acids 76–123 of ABA with the corresponding residues of APA A chain. Each box represents one recombinant clone obtained where the ABA residues highlighted in red are mutated to the corresponding amino acids of APA A chain, also highlighted in red. (C) Mutants of the truncated chimeric protein ABA_1–123_APA_124–175_ namely, T_82_Q_83_H_85_ to SEF, L_87_D_89_ to FN, S_92_D_96_ to AT and D_103_H_105_ to QY bound the mAb D6F10 whereas the mutants namely, Y_110_T_112_G_114_R_118_ to DNDK and T_112_G_114_R_118_ to NDK showed very little binding to the antibody.

The A chains of abrin and its homolog, *Abrus precatorius* agglutinin (APA) share 67% sequence identity and their crystal structures are very similar [34] but unlike abrin, APA does not bind the mAb D6F10 [20]. As further truncation analysis of ABA 1–175 was not possible, recombinant chimeric proteins (∼45 kDa) between ABA and APA A chain were generated to delineate the epitopic region. Immunoblot analysis of the chimera APA_1–123_ABA_124–175_ with the mAb D6F10 revealed that substitution of the first 123 amino acids of ABA with the corresponding amino acids of APA resulted in loss of antibody binding (Figure 2A). Further, antibody binding of the chimera ABA_1–123_APA_124–175_ (equivalent to native abrin) indicated that amino acids 1–123 of ABA contain the epitope of mAb D6F10. Furthermore, amino acids 74–123 harbour the core epitope of mAb D6F10 as the antibody did not bind the chimeric protein ABA_1–73_APA_74–175_, wherein, these amino acids were swapped by the corresponding residues of APA A chain (Figure 2A). An increase in antibody binding was also observed when the region 74–123 of APA A chain full-length protein was swapped to the corresponding ABA residues (Figure S1). Sequence alignment of ABA and APA A chain revealed that 13 residues were different within the stretch of amino acids 74–123 corresponding to ABA (Figure 2B). Immunoblot analysis of the site-directed mutants of these residues namely, T_82_Q_83_H_85_ to SEF, L_87_D_89_ to FN, S_92_D_96_ to AT, D_103_H_105_ to QY, Y_110_T_112_G_114_R_118_ to DNDK and T_112_G_114_R_118_ to NDK in the chimera ABA_1–123_APA_124–175_ (∼45 kDa) revealed that Thr112, Gly114 and Arg118 are crucial for the formation of the epitope of mAb D6F10 as mutation of these residues resulted in loss of antibody binding (Figure 2C).

### Effect of mAb D6F10 on Cytotoxicity of Abrin

**Figure 3:**
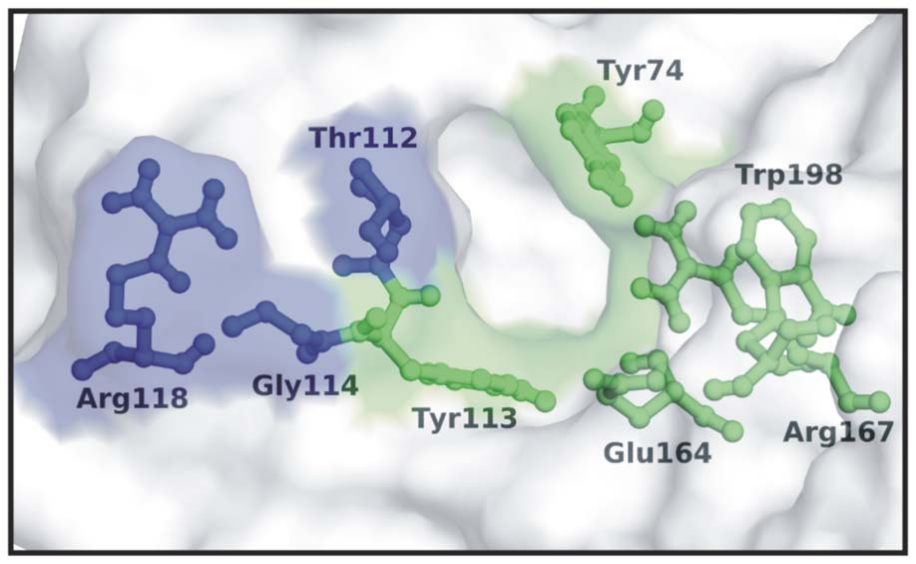
The epitope corresponding to the mAb D6F10 is present close to the active site cleft of ABA. Surface diagram of ABA showing the active site cleft. The residues Tyr74, Tyr113, Glu164, Arg167 and Trp198 (in green) are the active site residues of ABA whereas the amino acids Thr112, Gly114 and Arg118 (in blue) are involved in the formation of the epitope. The figure clearly illustrates that Thr112 and Gly114 residues are present very close to the active site cleft of ABA.

**Figure 4:**
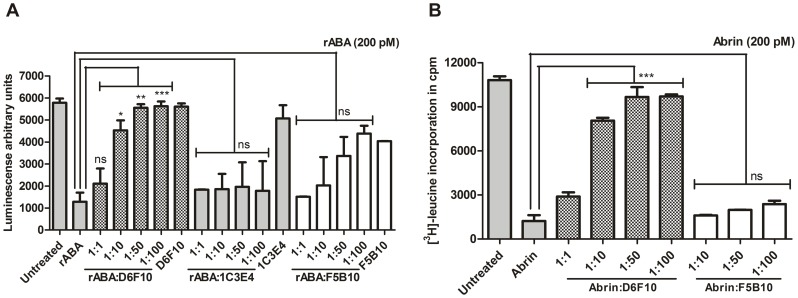
The mAb D6F10 blocks abrin’s enzymatic activity/cytotoxicity. (A) Rescue of inhibition of protein synthesis by recombinant ABA (rABA) in cell-free system. Rabbit reticulocyte lysate containing amino acids and luciferase mRNA was treated with either 200 pM recombinant ABA (rABA) alone or in the presence of varying molar concentrations of the mAbs D6F10, F5B10 or 1C3E4 for 1 h at 37°C followed by addition of the luciferin substrate. The rescue of inhibition of protein synthesis in the test samples by the mAb D6F10 was evaluated as the luminescence obtained in comparison with the rABA control. The figure represents data obtained from three different experiments. (B) Rescue of inhibition of protein synthesis by abrin in HeLa cells. 0.2 million HeLa cells were cultured with either 200 pM abrin alone or in the presence of varying molar concentrations of the mAbs D6F10 or F5B10 for 7 h, starved in leucine-free RPMI for 2 h and pulsed with [^3^H] leucine for 1 h. Rescue of inhibition of protein synthesis by mAb D6F10 in abrin treated cells was evaluated as the radioactivity incorporated into test samples compared with the untreated control. The assay was performed in duplicates and carried out three times. Statistical analysis was performed using One way ANOVA followed by Tukey’s multiple comparison tests (*p<0.05).

Analysis of the spatial position of the amino acids involved in the formation of the epitope (Thr112, Gly114 and Arg118) revealed that they are present in close proximity to the active site cleft/substrate binding site of ABA (Figure 3). Therefore, binding of the mAb D6F10 might physically obstruct the access of the substrate of ABA (adenine 4324 of *α*-sarcin loop) [35] into its active site cleft, thereby resulting in the rescue of inhibition of protein synthesis (PSI) by ABA. The hypothesis was confirmed using a cell-free luciferase assay system, wherein, significant (p<0.05) rescue of PSI was observed by addition of the mAb D6F10 along with recombinant ABA (Figure 4A). Another antibody against ABA namely, F5B10, known to be non-neutralizing in cells and mice [20] appeared to partially rescue PSI *in vitro*, although the observation was not found to be statistically significant, as determined by One way ANOVA followed by Tukey’s multiple comparison test. An unrelated isotype control antibody, 1C3E4 did not show any rescue of PSI (Figure 4A). Furthermore, in HeLa cells, a dose dependent rescue of PSI was observed with mAb D6F10 but not with mAb F5B10 (Figure 4B), as reported earlier for other cell lines [20].

### Prevention of Cell Surface Binding of Abrin by the Antibody

**Figure 5:**
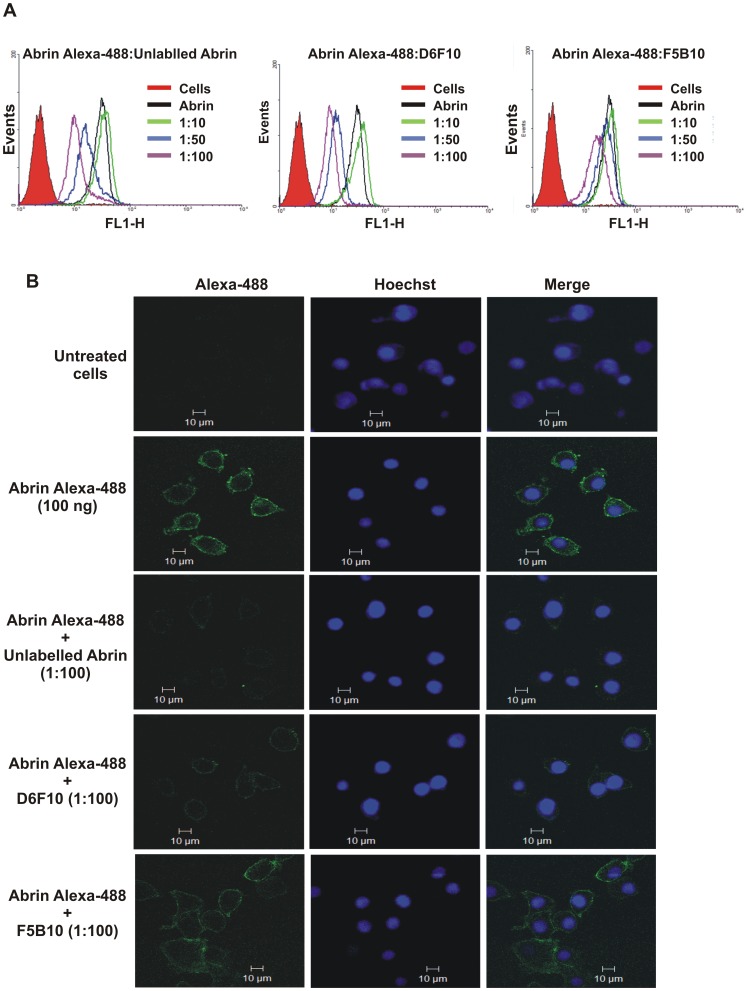
The mAb D6F10 reduces the binding of abrin on HeLa cells at high concentrations. (A) 500 ng/ml of Alexa-488 labelled abrin was incubated with varying molar concentrations of the mAbs D6F10 or F5B10 or unlabelled abrin for 1 h at 4°C. 0.2 million HeLa cells were treated with the pre-incubated samples for 1 h on ice, washed with ice cold PBS and analysed by FACScan. Significant reduction in cell attachment of abrin was observed with unlabelled abrin (positive control) and mAb D6F10 (at 50 and 100 fold molar concentration) but not with F5B10 (isotype control). The figure is a representative of three separate experiments performed. (B) 0.04 million HeLa cells adhered on a cover slip were treated with Alexa-488 labelled abrin in the presence of 100 fold molar excess of unlabelled abrin, mAbs D6F10 or F5B10 for 1 h. Cells were then fixed with paraformaldehyde, stained with Hoechst dye, mounted on slides and observed under a Zeiss confocal scanning microscope.

Earlier studies have reported that the mAb D6F10 prevented binding of abrin to Jurkat cells [20]. The prevention of cell surface binding of abrin by the mAb D6F10 was confirmed in HeLa cells to ensure that the previous observation was not cell-specific. Binding of Alexa-488 labelled abrin on the surface of HeLa cells was found to decrease only in the presence of high concentrations (50 and 100 fold molar excess) of the mAb D6F10 but the effect was only marginal for the isotype control antibody F5B10 (Figure 5A). These results were reconfirmed using confocal microscopy, wherein, cell surface binding of Alexa-488 labelled abrin was analysed in the presence of 100 fold molar excess of mAb D6F10 (Figure 5B). Surprisingly, though rescue of PSI was observed at the abrin:mAb molar concentration of 1∶10, no inhibition of binding of abrin to cells was seen in flow cytometric analysis (Figure 4 and 5A). Moreover, the presence of the neutralizing epitope close to the active site of abrin and rescue of PSI in cells in the absence of inhibition of cell attachment of abrin highlighted the possibility that the mAb D6F10 might rescue abrin-mediated toxicity intracellularly.

### Mechanism of Neutralization of Abrin

**Figure 6:**
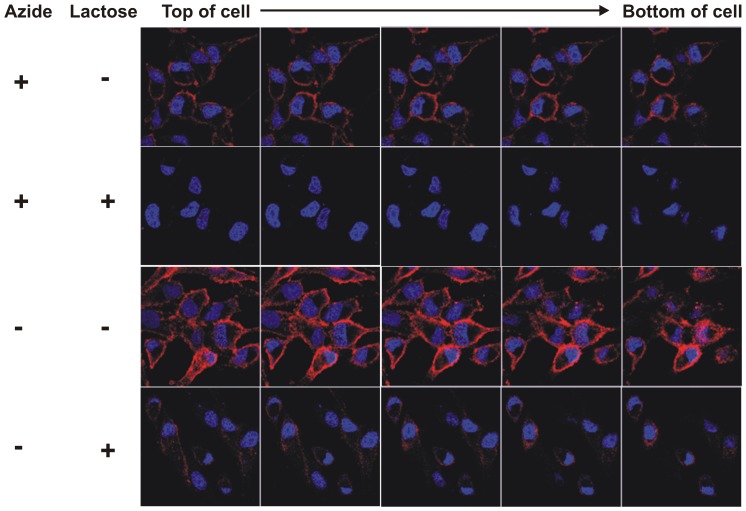
The mAb D6F10 binds and internalizes in HeLa cells along with abrin. 0.04 million HeLa cells adhered on a cover slip were incubated with 1∶50 dilution of normal goat serum in complete DMEM for 1 h at 37°C or 4°C with 0.01% azide. Cells were then incubated for 45 min with 100 ng abrin and 10 fold molar excess of the antibody, washed twice with PBS with/without lactose and fixed with paraformaldehyde. After two washes with PBS, cells were incubated with 1∶500 dilution of anti-mouse Alexa-633 labelled secondary antibody for 1 h at room temperature. Following this, the cells were stained for their nuclei with propidium iodide (represented as blue color) containing RNase, washed again and mounted on slides. The stained cells were observed under a Zeiss confocal scanning microscope and Z-stacks separated by 0.75 µm were imaged.

To examine the possibility that the mAb D6F10 might function intracellularly, we analysed the internalization of the antibody in HeLa cells by confocal microscopy. Cell surface binding of the antibody was observed when incubated with abrin at 4°C in the presence of azide (Figure 6). The binding was completely abolished on washing the cells with 0.2 M lactose confirming that the mAb D6F10 localizes to the cell membrane by binding to abrin. However, at 37°C in the absence of azide, cell surface binding and internalization of the antibody was observed in cells. Furthermore, removal of the surface bound abrin-antibody complex by lactose clearly showed the presence of the internalized antibody in cells (Figure 6). The internalization of the abrin-antibody complex was also observed in HeLa cells using directly labelled proteins. Alexa-488 labelled abrin (positive control) clearly showed cell surface binding of the toxin to cells and internalization of the same (Figure S2). In contrast, Alexa-633 labelled mAb D6F10 alone neither bound nor internalized in the cells. However, when cells were incubated with the labelled antibody in the presence of unlabelled abrin, clear signal of red fluorescence indicated the presence of the antibody bound on the cell surface and also inside cells. Colocalization of the antigen-antibody complex was also observed inside the cells with 1∶10 molar concentration of Alexa-488 labelled abrin:Alexa-633 labelled mAb D6F10 (Figure S2). The fact that prevention of cell attachment of abrin was not observed with ten fold molar excess of the antibody whereas, rescue of PSI and internalization of abrin and the antibody was observed suggested that the mAb D6F10 might rescue abrin-mediated PSI intracellularly (Figure 4, 5A and 6). It can be speculated that the antibody interferes with the intracellular trafficking of abrin or directly blocks its catalytic activity by physically occluding its active site.

## Discussion

D6F10 is the only known neutralizing antibody against abrin and has been shown to rescue toxicity of abrin in human cell lines as well as in mice [20]. To understand the mechanism of neutralization of abrin’s toxicity by the antibody, studies were initiated to map the epitope corresponding to the mAb D6F10. The antibody was shown to bind native abrin subjected to denaturing conditions [20] suggesting that the epitope might be linear/sequential. In contrast, this study reveals that the epitope on ABA is conformation-dependent. Reduced binding of the truncated protein ABA 76–175 in comparison to full-length ABA or the chimera ABA_1–123_APA_124–175_ suggested that the epitope is not linear. Also, absence of binding of the truncated proteins of ABA spanning amino acids 1–130, 1–143 or 1–160 to the mAb D6F10 confirmed that the epitope is conformation-dependent. Further, the amino acid sequence 1–175 of ABA, comprising almost the first two domains of the protein, is necessary for attaining the appropriate conformation required for the binding of the mAb D6F10 (Figure 1). It is possible that at least partial refolding of these domains on the nitrocellulose membrane results in antibody binding, as reported earlier for certain other proteins [36]. Majority of the epitopes corresponding to neutralizing antibodies against ricin A chain have also been shown to be present within the first two domains of the protein and 88% of the neutralizing epitopes were found to be conformation-dependent/discontinuous [24]. A known ricin A chain (RTA) vaccine namely, RTA 1–33/44–198 with its C-terminal domain truncated has also been reported [37]. Thus, the present study on abrin is consistent with earlier findings on ricin. Thus superior vaccine candidates based on minimum essential folding domains required for binding to neutralizing antibodies could be designed against these toxins [38]. Furthermore, it was found that amino acids 74–123 of ABA contain the core epitope corresponding to the mAb D6F10 and the residues Thr112, Gly114 and Arg118 of ABA are important for binding of the antibody (Figure 2 and S1). As identification of discontinuous epitopes is difficult, very few neutralizing epitopes have been reported till date [39]. Epitopes on ricin A chain have been mapped using phage display libraries [40], where the cross reacting peptides only mimic the core interaction site of the epitopic region [41]. This study, to our knowledge, is the first report on a neutralizing epitope of the abrin toxin and the residues important for antibody binding have also been delineated.

The neutralizing epitope was found to be present in close proximity to the active site of ABA indicating that the binding of the antibody to the epitope would possibly block the enzymatic activity of ABA (Figure 3). This hypothesis was confirmed as the mAb D6F10 rescued the inhibition of protein synthesis by ABA in a dose-dependent manner in a cell-free system (Figure 4A). The rescue of PSI was also observed in cells at varying concentrations of the antibody (Figure 4B). In concordance with the earlier report [20], it was observed that high concentrations of the mAb D6F10 prevents cell attachment of abrin (Figure 5). Antibodies against several toxins are known to protect by blocking their entry in cells [28], [42]. However, the precise mechanism by which anti-A chain antibodies sterically hinder the binding of the B chain to the cell surface still remained unclear [30]. *In silico* analysis of the position of the mapped epitope of the mAb D6F10 on the structure of abrin revealed that the epitope lies far from the galactose binding pockets of the B chain (Figure S3). Thus, it is unlikely that binding of the antibody to the A chain of abrin would sterically hinder the binding of the B chain to the cell surface, as was hypothesized earlier for mAb D6F10 and anti-A chain antibodies to ricin [30]. Moreover, prevention of abrin binding to cells was observed only with high concentrations of the antibody (Figure 5A) whereas, the rescue of PSI was also observed with 10 fold molar excess of the antibody (Figure 4). Furthermore, cell surface binding and internalization of the mAb D6F10 was observed at 1∶10 molar concentration of abrin:antibody (Figure 6 and S2). The colocalization of the antigen-antibody complex in HeLa cells clearly suggested that mAb D6F10 binds the A chain of abrin and enters the cells. Thus, the mAb D6F10 possibly acts inside cells to prevent abrin’s toxicity. The neutralization observed in cells could be a result of altered trafficking of abrin in the presence of bound antibody or due to blockage of the enzymatic activity of abrin. The findings of the present study shed light on the possible underlying mechanisms of immunoneutralization of ricin by anti-ricin A chain antibodies [15]. Internalization of the anti-A chain antibody provides the missing link between the observed block of the RNA N-glycosidase activity of the A chain in cell-free system and neutralization of these toxins in whole cells and animals [21]. Furthermore, it might explain the reason for higher efficacy of neutralization of ricin by anti-A chain antibodies in comparison to the anti-B chain antibodies, which are known to interfere with cell attachment [29], [31], [40]. These results are also consistent with earlier findings wherein neutralizing antibodies were shown to interfere with protein synthesis whereas non-neutralizing antibodies did not [24]. A very recent article by Song *et al.* described an antibody to ricin A chain that gets internalized in cells along with ricin and protects cells from ricin’s toxicity by hindering the intracellular routing of the toxin [43].

It was surprising that mAb D6F10 could allow binding and internalization of abrin at 1∶10 molar concentration of abrin:antibody (Figure 6) but prevented cell attachment of abrin only at higher molar concentrations (Figure 5). Taken together, our results suggest that abrin:mAb D6F10 present at lower ratios, i.e., 1∶10, form small immune complexes that can get internalized in the cell and abrin toxicity is prevented. However, at higher abrin:mAb ratios (1∶50, 1∶100), the cell surface binding of abrin is reduced suggesting that the immune complexes formed are possibly larger, thereby sterically hindering cell surface binding of abrin (Figure S4). We speculate that the contradictory effects of increasing doses of the antibody on binding of abrin to cells could be explained by the fact that antibodies are glycoproteins and possess branched sugars (on the CH2 constant domain in case of IgG) which may end in terminal galactose or its derivative N-acetyl galactosamine [44]. The binding of branched glycans of the antibody to the galactose binding pocket of the B chain of abrin might come into play at higher concentrations of the antibody. This would prevent binding of abrin to cells by either blocking the galactose binding pocket of the B chain directly or forming huge complexes of the antibody and abrin which cannot internalize in cells (Figure S4). Studies are underway to test the speculated model and unravel the trafficking of the antigen-antibody complex inside the cell.

## Materials and Methods

### Cells

HeLa cells were obtained from Vrije University hospital, Amsterdam and cultured in Dulbecco’s modified Eagle’s medium (DMEM, Sigma-Aldrich) supplemented with 10% fetal bovine serum (FBS, PAN Biotech), 100 I.U/ml penicillin, 100 µg/ml streptomycin and 5 µg/ml nystatin (complete medium). D6F10 and F5B10 hybridoma cells [20] were cultured in Iscove’s modified Dulbecco’s medium (IMDM) supplemented with 10% FBS, antibiotics and 50 µM *β*-mercaptoethanol. The cultures were maintained at 37°C in a humidified 5% CO_2_ incubator and passaged every 3–4 days.

### Plant Proteins and Antibodies

The native plant proteins, abrin and *Abrus precatorius* agglutinin (APA) were purified from matured seeds of *Abrus precatorius* as described earlier [34], [45]. Monoclonal antibodies from the culture supernatants of hybridoma cells were purified by Protein A-Sepharose affinity chromatography [46]. The purity of the mAbs was confirmed by subjecting the purified samples to polyacrylamide gel electrophoresis followed by Coomassie blue staining (Figure S5). Abrin and the mAb D6F10 were labelled with Alexa-488 and Alexa-633 dye (Invitrogen, USA) respectively [47].

### Bioinformatic Analysis

Visualization of ABA structure (PDB code-1ABR) and generation of structural images was performed using open-source PyMOL software (www.pymol.org). Sequence alignment of abrin and APA was carried out using BLASTp [48]. GenBank accession number used for abrin and APA are M98344.1 and AF190173 respectively.

### Cloning of Deletion Constructs of ABA

Deletion constructs of ABA were generated using the full-length ABA gene as the template. The constructs were cloned in frame with the N-terminal GST tag in the pGEX5X2 vector between *Bam*HI and *Xho*I restriction enzyme sites.

### Generation of Chimeric Constructs of Abrin and APA A Chains

The numbering of the APA A chain constructs and its chimeric constructs is in accordance to the sequence alignment with ABA. The quick-change method (Stratagene, USA) was employed to introduce a silent substitution at codon 123 of ABA for incorporation of *Sal*I restriction site in the ABA 1–175 construct. APA A chain gene constructs namely, APA 1–123 and APA 124–175 were acquired from Genscript, USA, cloned in pUC57 vector between *Bam*HI/*Sal*I and *Sal*I/*Xho*I restriction enzyme sites respectively. The chimeric constructs APA_1–123_ABA_124–175_ and ABA_1–123_APA_124–175_ were cloned by insertion of the APA 1–123 and 124–175 sequence into the ABA 1–175 *Sal*I mutant using *Bam*HI/*Sal*I and *Sal*I/*Xho*I sites respectively. The chimeric construct ABA_1–73_APA_74–175_ was generated using the *Nde*I restriction site present at codons 73–74 in both ABA and APA A chain. Codons 74–123 of ABA_1–123_APA_124–175_ were replaced by APA 74–123 sequence using *Nde*I/*Sal*I digestion to obtain the ABA_1–73_APA_74–175_ construct. Similarly, an APA A chain full-length chimeric construct (APA chimera) was also cloned by replacing codons 74–123 of APA with those corresponding to ABA using *Nde*I/*Sal*I sites. All the chimeric constructs were cloned in frame with the N-terminal GST tag in the pGEX5X2 vector. Site-directed mutant genes were generated on the chimera ABA_1–123_APA_124–175_ either by quick-change method or overlap-extension method [49] and confirmed by sequencing.

### Expression and Immunoblot Analysis of the Recombinant Proteins

*E. coli* BL21 (DE3) pLysS cells were transformed with the different recombinant plasmids for expression of the recombinant proteins. The primary inoculum was grown overnight at 37°C followed by secondary inoculum at 30°C till the culture reached 0.6 OD (at 600 nm) after which it was induced with 400 µM IPTG (isopropyl-β-D-thiogalactopyranoside) for 6–8 hrs at 16°C. Uninduced cultures were used as negative control for the experiments. *E. coli* cells containing the desired expressed protein were electrophoresed on a 12.5% polyacrylamide gel under reducing conditions and transferred on to nitrocellulose membrane. The membrane was blocked (0.2% BSA in PBS, pH 7.2) for 2 h followed by incubation with the hybridoma culture supernatant containing the respective primary antibody (anti-GST or mAb D6F10) for 2 h. Further, the membrane was washed, incubated with the secondary antibody (rabbit α-mouse Ig-HRP, Dako, Denmark) for 1 h, washed again and developed using diaminobenzidine (DAB) or ECL kit (Millipore). Immunoblot analysis with GST-specific antibody confirmed presence of the GST-fusion proteins of expected molecular sizes. Native abrin (A chain, ∼30 kDa) served as the positive control for D6F10 blot and GST-perforin fusion protein (GST+, ∼45 kDa) for the GST blot.

### Protein Synthesis in Cell-free System

Rabbit reticulocyte lysate (RRL kit, Promega) cocktail was prepared containing total amino acids, RNase inhibitor and luciferase mRNA. 200 pM of either ABA alone or with the mAbs D6F10, F5B10 or 1C3E4 (IgG isotype control) were prepared in 0.25 µl and incubated with 9.75 µl of the RRL cocktail for 1 h at 37°C. Protein synthesis was quantified by the luminescence obtained by adding 1 µl of the reaction mixture to 30 µl of luciferin substrate. The recombinant abrin A chain used for this assay was expressed in *E. coli* (DE3) BL21 pLysS cells and purified by Ni-NTA affinity chromatography [20].

### Protein Synthesis in HeLa Cells

0.2 million HeLa cell/250 µl complete medium/well were plated in a 24 well plate and allowed to adhere. Cells were incubated for 7 h with abrin alone or abrin pre-incubated with increasing molar excess of the mAbs D6F10 or F5B10 for 1 h. The cells were washed twice with phosphate buffer saline (PBS), incubated with 200 µl of leucine-free RPMI 1640 medium (US Biological) for 2 h and then pulsed with 0.4 µCi of [^3^H] leucine (BRIT, India) for 1 h. 5% trichloroacetic acid was added to precipitate the proteins and kept overnight at 4°C. The precipitated proteins were washed twice with 20% ethanol, dried, dissolved in 200 µl of 1% SDS in 0.1 N NaOH and incubated with 4 ml of scintillation cocktail overnight. The radioactivity was measured in a scintillation counter (Beckman Coulter).

### Fluorescence Activated Cell Scan (FACScan) Analysis

0.2 million HeLa cells were harvested and washed twice with ice cold PBS. 500 ng/ml of Alexa-488 labelled abrin alone or labelled abrin with the mAbs was incubated for 1 h at 4°C (total volume 200 µl). Cells were incubated with the samples for 1 h on ice, washed twice with ice cold PBS and analyzed by FACScan (Becton Dickinson).

### Confocal Microscopy

0.04 million HeLa cells in 100 µl complete DMEM were allowed to adhere on cover-slips overnight. The Fc receptors on the cells were blocked with normal goat serum (at 1∶50 dilution) in 100 µl DMEM for 1 h. Half the cover-slips were blocked at 37°C whereas the other half at 4°C with 0.01% azide to inhibit cell surface internalization. Cells were treated with abrin and the mAb D6F10 present at 1∶10 molar ratio for 45 min either at 37°C or 4°C with 0.01% azide. After the treatment, cells were washed twice (5 min each) with PBS with/without 0.2 M lactose, fixed with 4% paraformaldehyde, washed twice with PBS followed by incubation with 1∶500 dilution of anti-mouse Alexa-633 labelled secondary antibody (Invitrogen, USA) for 1 h at room temperature. The nucleus in cells was stained using 50 µg/ml propidium iodide in the presence of 10 µg/ml RNase for 30 min. Cells were again washed and mounted on slides using 10 µl mounting solution (90% glycerol containing Mowiol anti-fade, Calbiochem, USA). The slides were visualized and images acquired using Zeiss LSM 510 Meta Laser Confocal scanning microscope (Carl Zeiss Foundation, Germany) and the images were further produced using the Zeiss LSM image browser. Z-stacks with 0.75 µm steps were acquired to visualize the internalized antibody. For the microscopy studies presented in figures 5B and S2, cells were blocked with normal mouse serum and incubated with different reactants (100 ng Alexa-488 labelled abrin with 10 fold molar excess of Alexa-633 labelled mAb D6F10 or 100 fold molar excess of unlabelled D6F10) in 100 µl of 0.2% BSA in PBS for 1 h at room temperature. Cells were washed, fixed with paraformaldehyde and stained with 5 µg/ml of Hoechst-33342 dye for 7 min.

## Supporting Information

Figure S1 **Amino acid sequence 74–123 comprise the core epitope on ABA.** The uninduced (Un) and induced (In) samples of the chimeric proteins (∼45 kDa) of ABA and APA A chain were subjected to immunoblot analysis with mAb D6F10 or anti-GST antibody. The recombinant APA A chain full-length did not bind the mAb D6F10 unlike abrin (positive control). The APA A chain chimera (amino acids 74–123 swapped by corresponding residues of ABA) showed weak binding to the mAb D6F10.
(TIF)

Figure S2 **The mAb D6F10 binds and internalizes in HeLa cells along with abrin.** 0.04 million HeLa cells adhered on a cover slip were incubated with 1∶50 dilution of normal mouse serum in DMEM for 1 h. Cells were then incubated for 1 h with different samples at room temperature. After the treatments, cells were fixed with paraformaldehyde, washed twice with PBS, stained with Hoechst dye and mounted on slides. The stained cells were observed under a Zeiss confocal scanning microscope. Cell surface binding and internalization of the antigen-antibody complex was observed in HeLa cells when used at 1∶10 molar concentration.
(TIF)

Figure S3 **The mapped epitope corresponding to mAb D6F10 is spatially far from the B chain of abrin.** The ABA is represented in green, the B chain is coloured blue and the residues Thr112, Gly114 and Arg118 (crucial for binding to mAb D6F10) are represented as red sticks. The figure illustrates that the epitope lies far from the functional domains of the B chain of abrin.
(TIF)

Figure S4 **Proposed model for immunoneutralization of abrin by the mAb D6F10.** (A) At 1∶10 molar ratio of abrin:mAb D6F10 the antigen-antibody complex binds to the surface of HeLa cells and internalizes into the same. Thus inhibition of protein synthesis by abrin is blocked intracellularly by the bound antibody either by interfering with the toxin transport or binding close to the active site cleft of ABA. (B) At 100 fold molar excess of the mAb D6F10, the binding of the glycans of the antibody to the galactose binding pocket of the B chain of abrin might come into play. This could either block the binding of B chain to the cell surface through its galactose binding pocket or lead to formation of huge antigen-antibody complexes (encircled) which might not bind to cell surface.
(TIF)

Figure S5 **The mAb D6F10 is pure and free of any contaminating protein.** 20 and 40 µg of the purified mAb D6F10 was electrophoresed on a 12.5% polyacrylamide gel under reducing conditions and stained with Coomassie blue to visualize the protein bands.
(TIF)
