# Supplementary figures and images for: Mechanistic Insights into the Neutralization of Cytotoxic Abrin by the Monoclonal Antibody D6F10
Source: PLoS One. 2013 Jul 29;8(7):e70273. doi: 10.1371/journal.pone.0070273 (PMC3726390; doi:10.1371/journal.pone.0070273)

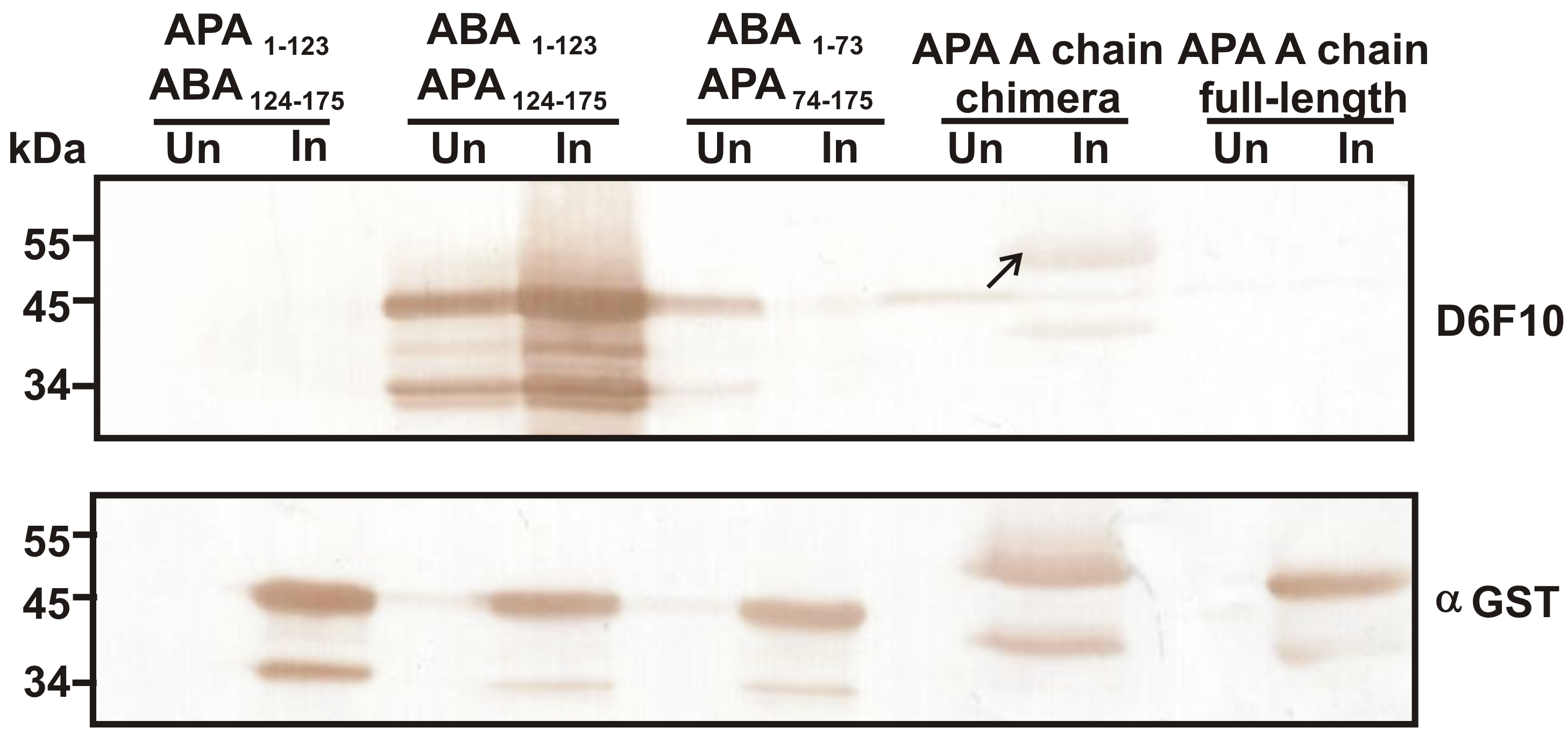

Supplement: Figure S1 — Amino acid sequence 74–123 comprise the core epitope on ABA. The uninduced (Un) and induced (In) samples of the chimeric proteins (∼45 kDa) of ABA and APA A chain were subjected to immunoblot analysis with mAb D6F10 or anti-GST antibody. The recombinant APA A chain full-length did not bind the mAb D6F10 unlike abrin (positive control). The APA A chain chimera (amino acids 74–123 swapped by corresponding residues of ABA) showed weak binding to the mAb D6F10. (TIF) [file pone.0070273.s001.tif]

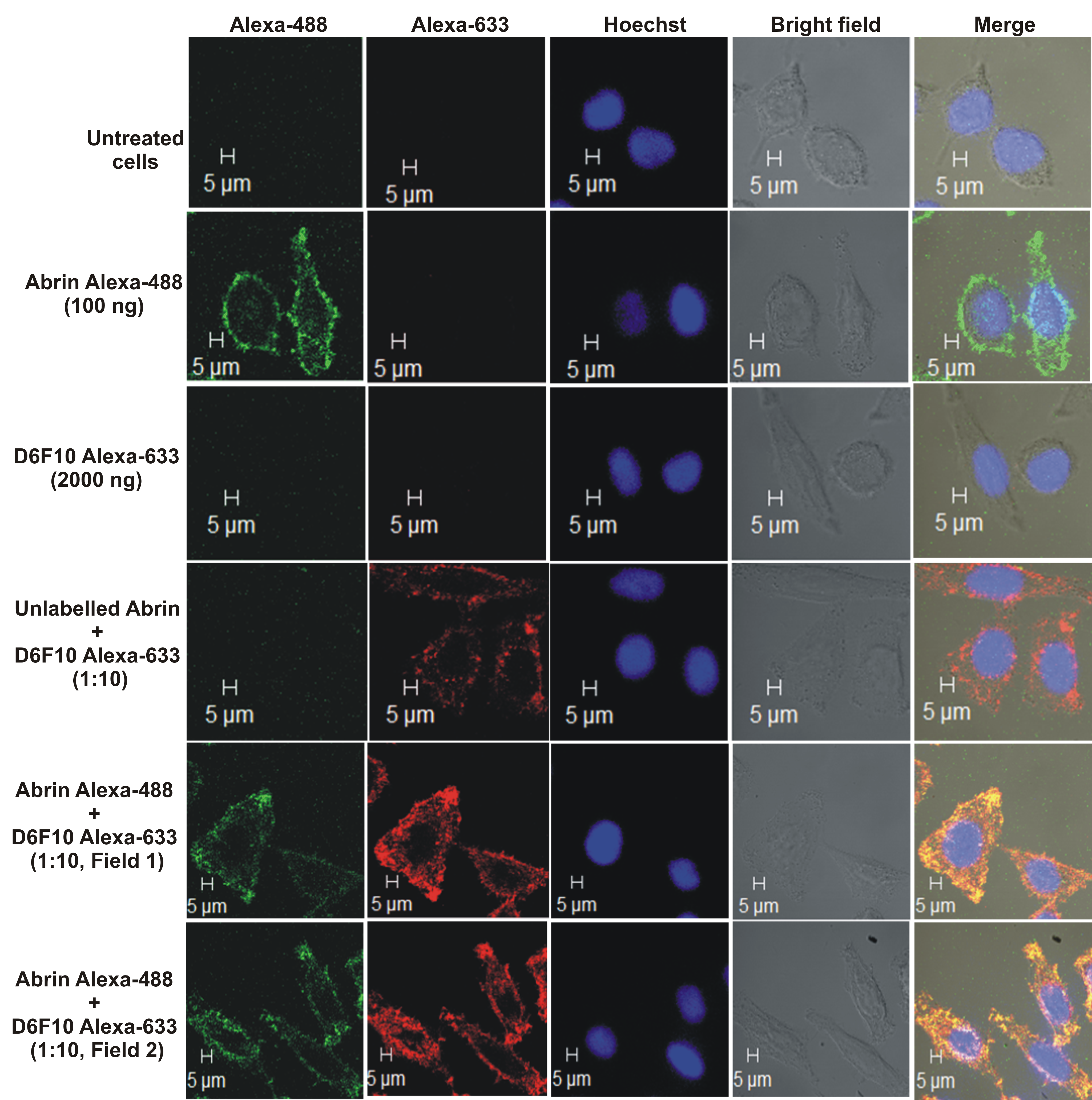

Supplement: Figure S2 — The mAb D6F10 binds and internalizes in HeLa cells along with abrin. 0.04 million HeLa cells adhered on a cover slip were incubated with 1∶50 dilution of normal mouse serum in DMEM for 1 h. Cells were then incubated for 1 h with different samples at room temperature. After the treatments, cells were fixed with paraformaldehyde, washed twice with PBS, stained with Hoechst dye and mounted on slides. The stained cells were observed under a Zeiss confocal scanning microscope. Cell surface binding and internalization of the antigen-antibody complex was observed in HeLa cells when used at 1∶10 molar concentration. (TIF) [file pone.0070273.s002.tif]

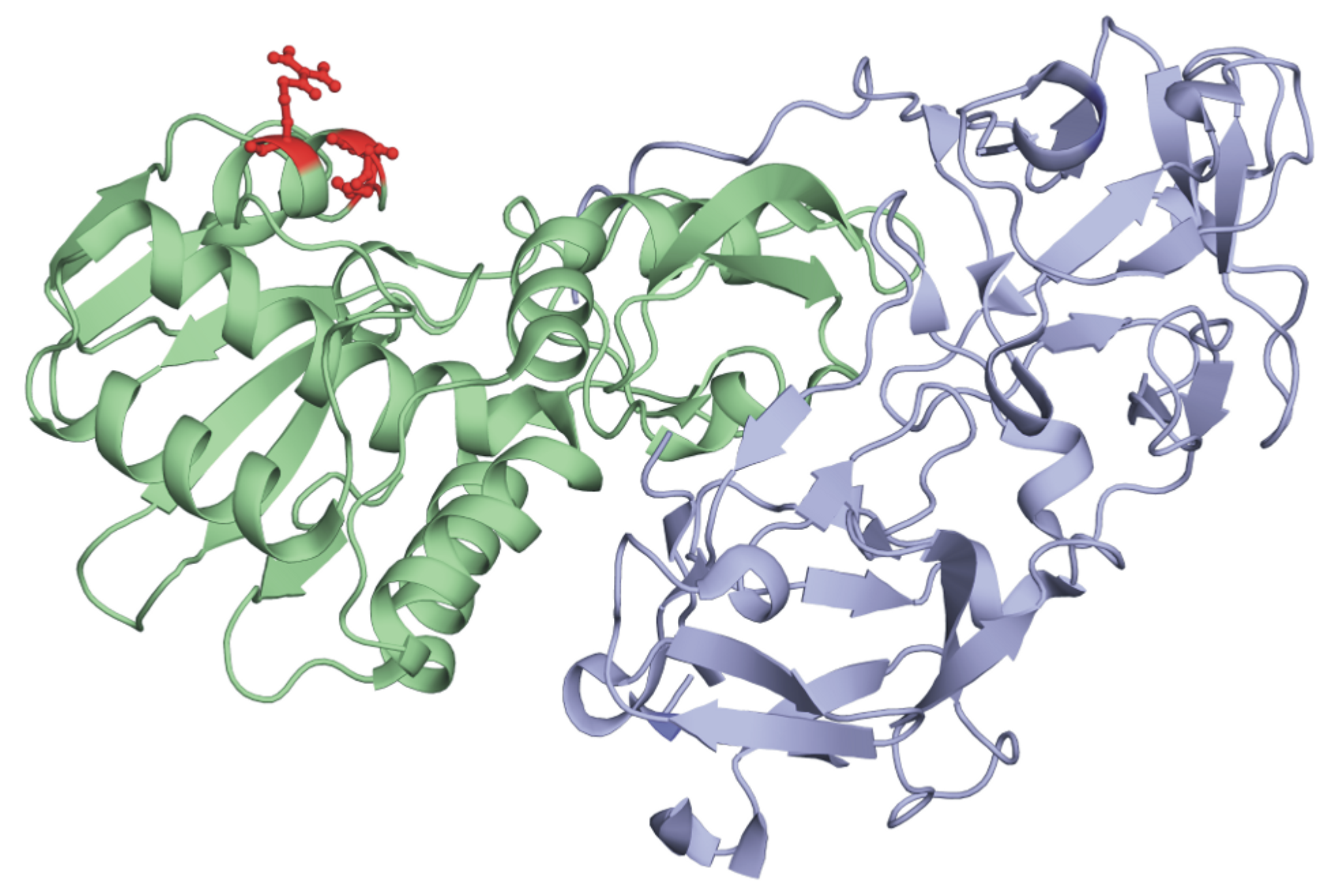

Supplement: Figure S3 — The mapped epitope corresponding to mAb D6F10 is spatially far from the B chain of abrin. The ABA is represented in green, the B chain is coloured blue and the residues Thr112, Gly114 and Arg118 (crucial for binding to mAb D6F10) are represented as red sticks. The figure illustrates that the epitope lies far from the functional domains of the B chain of abrin. (TIF) [file pone.0070273.s003.tif]

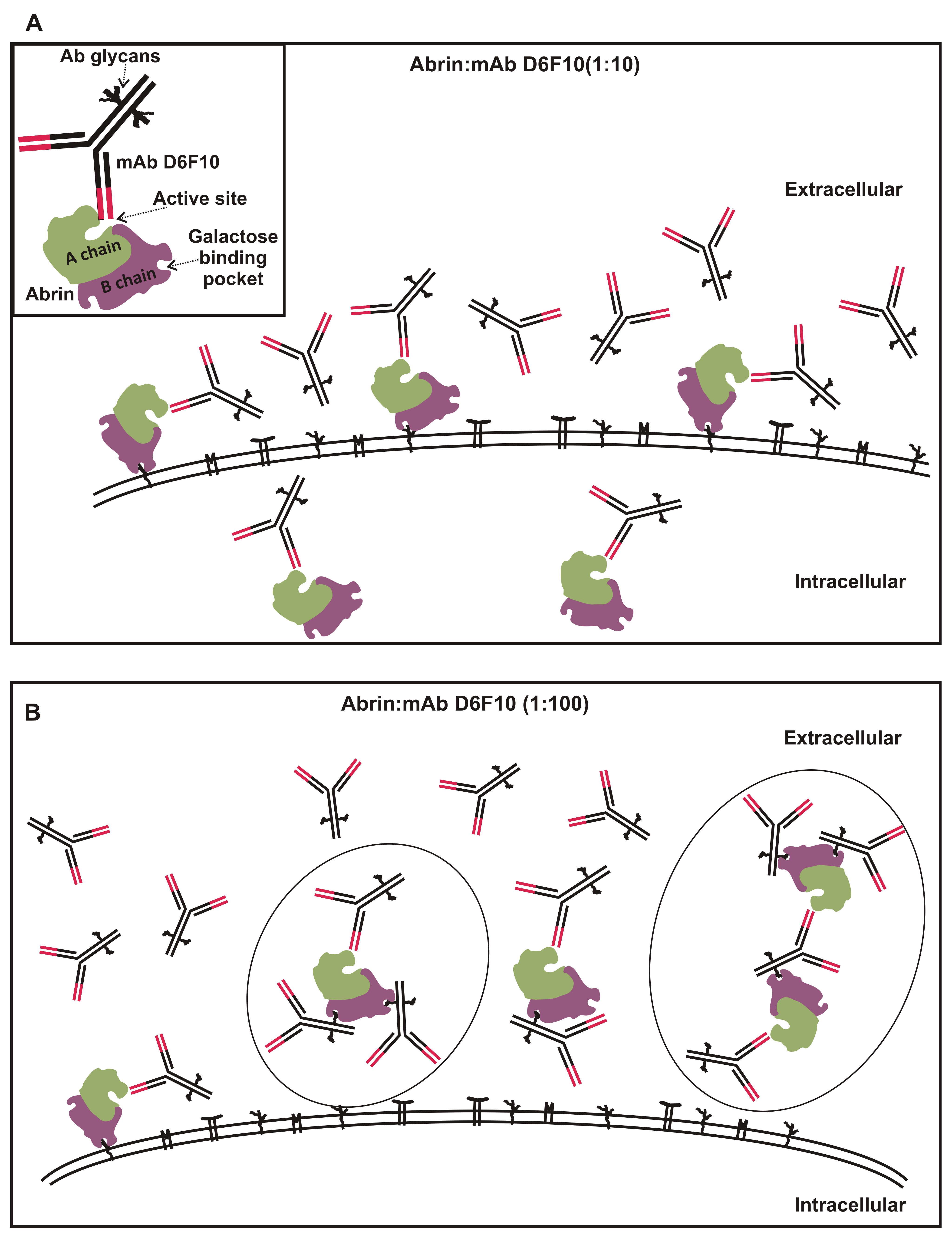

Supplement: Figure S4 — Proposed model for immunoneutralization of abrin by the mAb D6F10. (A) At 1∶10 molar ratio of abrin:mAb D6F10 the antigen-antibody complex binds to the surface of HeLa cells and internalizes into the same. Thus inhibition of protein synthesis by abrin is blocked intracellularly by the bound antibody either by interfering with the toxin transport or binding close to the active site cleft of ABA. (B) At 100 fold molar excess of the mAb D6F10, the binding of the glycans of the antibody to the galactose binding pocket of the B chain of abrin might come into play. This could either block the binding of B chain to the cell surface through its galactose binding pocket or lead to formation of huge antigen-antibody complexes (encircled) which might not bind to cell surface. (TIF) [file pone.0070273.s004.tif]

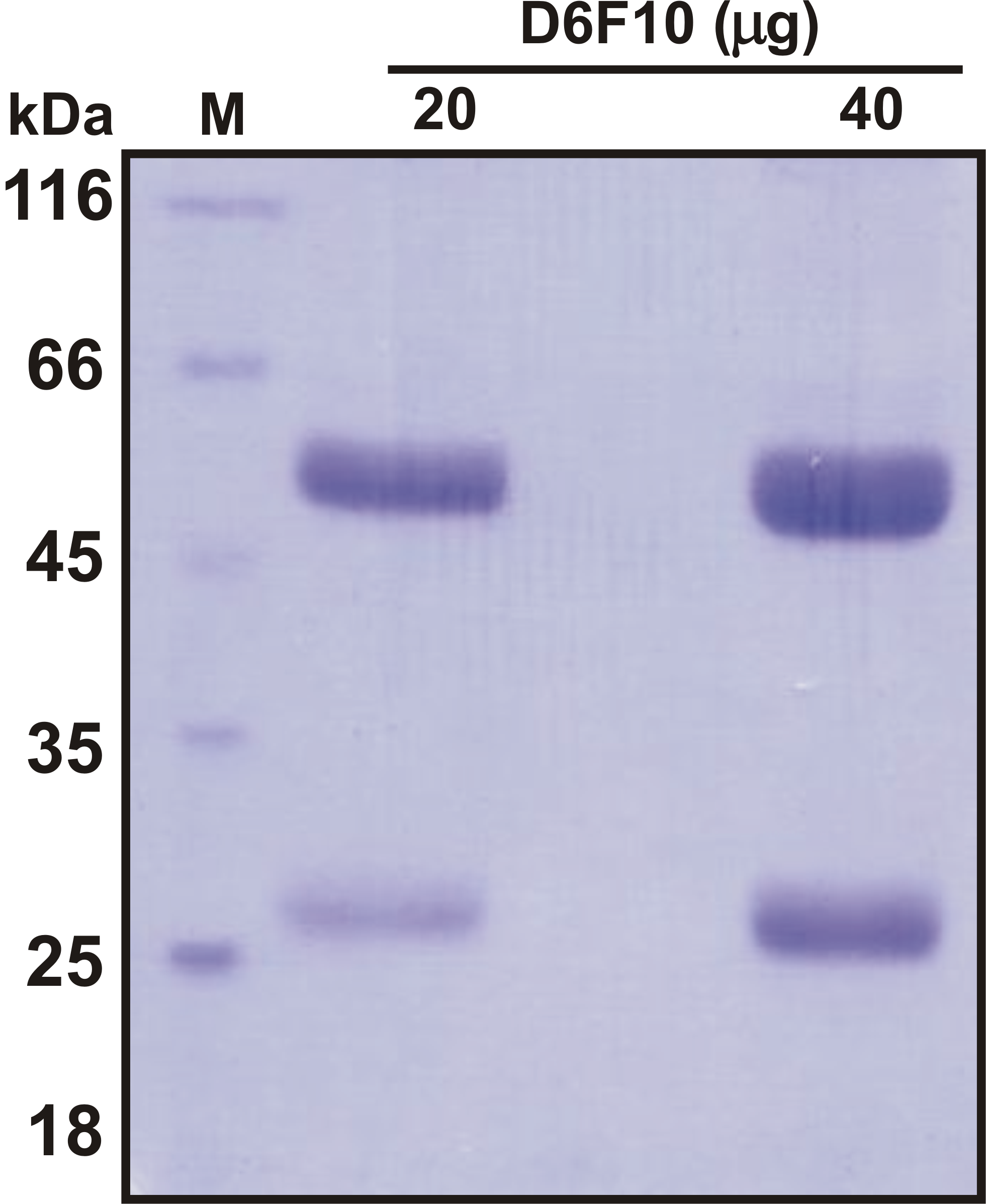

Supplement: Figure S5 — The mAb D6F10 is pure and free of any contaminating protein. 20 and 40 µg of the purified mAb D6F10 was electrophoresed on a 12.5% polyacrylamide gel under reducing conditions and stained with Coomassie blue to visualize the protein bands. (TIF) [file pone.0070273.s005.tif]
